# Prediction of methotrexate neurotoxicity using clinical, sociodemographic, and area-based information in children with acute lymphoblastic leukemia

**DOI:** 10.1093/oncolo/oyaf055

**Published:** 2025-06-23

**Authors:** Rachel D Harris, Olga A Taylor, Maria Monica Gramatges, Amy E Hughes, Mark Zobeck, Sandi Pruitt, M Brooke Bernhardt, Ashley Chavana, Van Huynh, Kathleen Ludwig, Laura Klesse, Kenneth Heym, Timothy Griffin, Rodrigo Erana, Juan Carlos Bernini, Ashley Choi, Yuu Ohno, Melissa A Richard, Alanna C Morrison, Han Chen, Bing Yu, Philip J Lupo, Karen R Rabin, Michael E Scheurer, Austin L Brown

**Affiliations:** Texas Children’s Hospital, Cancer and Hematology Center, Houston, TX, USA; Department of Pediatrics, Division of Hematology/Oncology, Baylor College of Medicine, Houston, TX, USA; Department of Pediatrics, Baylor College of Medicine, Center for Epidemiology and Population Health, Houston, TX, USA; Department of Epidemiology, Human Genetics, and Environmental Science, University of Texas Health Science Center at Houston, Houston, TX, USA; Texas Children’s Hospital, Cancer and Hematology Center, Houston, TX, USA; Department of Pediatrics, Division of Hematology/Oncology, Baylor College of Medicine, Houston, TX, USA; Department of Pediatrics, Baylor College of Medicine, Center for Epidemiology and Population Health, Houston, TX, USA; Texas Children’s Hospital, Cancer and Hematology Center, Houston, TX, USA; Department of Pediatrics, Division of Hematology/Oncology, Baylor College of Medicine, Houston, TX, USA; Peter O’Donnell Jr. School of Public Health, University of Texas Southwestern Medical Center, Dallas, TX, USA; Texas Children’s Hospital, Cancer and Hematology Center, Houston, TX, USA; Department of Pediatrics, Division of Hematology/Oncology, Baylor College of Medicine, Houston, TX, USA; Department of Pediatrics, Baylor College of Medicine, Center for Epidemiology and Population Health, Houston, TX, USA; Department of Epidemiology, Human Genetics, and Environmental Science, University of Texas Health Science Center at Houston, Houston, TX, USA; Peter O’Donnell Jr. School of Public Health, University of Texas Southwestern Medical Center, Dallas, TX, USA; Deparment of Pharmacy and Pharmaceutical Sciences, St. Jude Children’s Research Hospital, Memphis, TN, USA; Texas Children’s Hospital, Cancer and Hematology Center, Houston, TX, USA; Department of Pediatrics, Division of Hematology/Oncology, Baylor College of Medicine, Houston, TX, USA; Children’s Hospital of Orange County, University of California Irvine College of Medicine, Orange, CA, USA; Department of Pediatrics, University of Texas Southwestern Medical Center, Dallas, TX, USA; Department of Pediatrics, University of Texas Southwestern Medical Center, Dallas, TX, USA; Department of Hematology/Oncology, Cook Children’s Medical Center, Fort Worth, TX, USA; Texas Children’s Hospital, Cancer and Hematology Center, Houston, TX, USA; Department of Pediatrics, Division of Hematology/Oncology, Baylor College of Medicine, Houston, TX, USA; Vannie E. Cook Children’s Clinic, Cancer and Hematology Clinic, McAllen, TX, USA; Vannie E. Cook Children’s Clinic, Cancer and Hematology Clinic, McAllen, TX, USA; Texas Children’s Hospital, Cancer and Hematology Center, Houston, TX, USA; Department of Pediatrics, Division of Hematology/Oncology, Baylor College of Medicine, Houston, TX, USA; Texas Children’s Hospital, Cancer and Hematology Center, Houston, TX, USA; Department of Pediatrics, Division of Hematology/Oncology, Baylor College of Medicine, Houston, TX, USA; Texas Children’s Hospital, Cancer and Hematology Center, Houston, TX, USA; Department of Pediatrics, Division of Hematology/Oncology, Baylor College of Medicine, Houston, TX, USA; Department of Pediatrics, Baylor College of Medicine, Center for Epidemiology and Population Health, Houston, TX, USA; Department of Epidemiology, Human Genetics, and Environmental Science, University of Texas Health Science Center at Houston, Houston, TX, USA; Department of Epidemiology, Human Genetics, and Environmental Science, University of Texas Health Science Center at Houston, Houston, TX, USA; Department of Epidemiology, Human Genetics, and Environmental Science, University of Texas Health Science Center at Houston, Houston, TX, USA; Texas Children’s Hospital, Cancer and Hematology Center, Houston, TX, USA; Department of Pediatrics, Division of Hematology/Oncology, Baylor College of Medicine, Houston, TX, USA; Department of Pediatrics, Baylor College of Medicine, Center for Epidemiology and Population Health, Houston, TX, USA; Texas Children’s Hospital, Cancer and Hematology Center, Houston, TX, USA; Department of Pediatrics, Division of Hematology/Oncology, Baylor College of Medicine, Houston, TX, USA; Texas Children’s Hospital, Cancer and Hematology Center, Houston, TX, USA; Department of Pediatrics, Division of Hematology/Oncology, Baylor College of Medicine, Houston, TX, USA; Department of Pediatrics, Baylor College of Medicine, Center for Epidemiology and Population Health, Houston, TX, USA; Texas Children’s Hospital, Cancer and Hematology Center, Houston, TX, USA; Department of Pediatrics, Division of Hematology/Oncology, Baylor College of Medicine, Houston, TX, USA; Department of Pediatrics, Baylor College of Medicine, Center for Epidemiology and Population Health, Houston, TX, USA

**Keywords:** acute lymphoblastic leukemia, methotrexate, neurotoxicity, adverse events, pediatric

## Abstract

**Background:**

Methotrexate is a critical component of pediatric acute lymphoblastic leukemia (ALL) therapy that can result in neurotoxicity which has been associated with an increased risk of relapse. We leveraged machine learning to develop a neurotoxicity risk prediction model in a diverse cohort of children with ALL.

**Methods:**

We included children (age 2-20 years) diagnosed with ALL (2005-2019) and treated in Texas without pre-existing neurologic disease. Clinical information was obtained by medical record review. Neurotoxicity occurring post-induction and prior to maintenance therapy was defined as neurologic episodes occurring within 21 days of methotrexate. Suspected cases were independently confirmed by 2 pediatric oncologists. Demographic and clinical factors were compared using logistic regression. The dataset was randomly split (80/20) for training and testing. random forest (RF) with boosting and downsampling using 5-repeat, 10-fold cross-validation was used to construct a predictive model.

**Results:**

Neurotoxicity developed in 115 (8.7%) of 1325 eligible patients. Several factors including older age at diagnosis (OR = 1.19, 95% CI: 1.15-1.24) and Latino ethnicity (OR = 2.79, 95% CI: 1.83-4.35) were associated with neurotoxicity. The RF had an area under the curve of 0.77 with a train error rate of 0.29 and a test error rate of 0.24. The overall sensitivity was 0.73, and specificity was 0.69.

**Conclusions:**

In one of the largest studies of its kind, we developed a novel risk prediction model of methotrexate-related neurotoxicity. Ultimately, a validated model may help guide the development of personalized treatment strategies to reduce the burden of neurotoxicity in children diagnosed with ALL.

**Implications for Practice**

Methotrexate is a key component of central nervous system-directed chemotherapy in children and adolescents with acute lymphoblastic leukemia. However, methotrexate is associated with acute neurotoxicity, which may compromise the delivery of curative therapy. Risk factors for methotrexate neurotoxicity remain poorly defined. Therefore, we leverage machine learning methods to develop a clinical methotrexate neurotoxicity risk model. A model considering clinical and demographic factors achieved reasonable performance, but future models should consider additional genetic and non-genetic factors. The development of robust risk prediction models may inform the development and delivery of neurotoxicity prevention interventions to those at the highest risk.

## Introduction

Acute lymphoblastic leukemia (ALL) is the most commonly diagnosed malignancy in children, with a global annual age-adjusted incidence rate of 49 cases per 1 000 000 children.^1-3^ Due to improvements in risk-adapted therapy and supportive care, nearly 90% of children diagnosed with ALL in high-income countries are expected to survive at least 5 years after diagnosis.^1,4,5^ Methotrexate is a critical component of most contemporary pediatric ALL treatment protocols and plays an important role in the treatment and prevention of central nervous system (CNS) disease.^6^ However, intrathecal (IT) and intravenous (IV) methotrexate therapy results in acute neurotoxicity in 3% to 14% of children.^7-9^ Clinical manifestations of acute neurotoxicity can range from altered mental status to seizures, stroke-like symptoms, or encephalopathy.^7,10^ Although acute symptoms typically resolve within weeks, neurotoxicity often results in methotrexate treatment modifications and has been linked to an increased risk of ALL relapse.^8,9^ Despite being a significant source of morbidity and potentially compromising ALL treatment efficacy, risk factors for methotrexate-related neurotoxicity remain poorly defined.

Several clinical and demographic factors have been associated with the incidence of methotrexate-related neurotoxicity. In particular, older age at diagnosis and exposure to high-dose IV methotrexate (ie, 5 g/m^2^) have consistently been associated with an increased likelihood of neurotoxicity.^7-9,11,12^ Recent work also reported an association between peak serum transaminitis during the initial phases of ALL therapy and neurotoxicity risk.^9^ Finally, some evidence suggests Latino children are disproportionally affected by methotrexate-related neurotoxicity and clinical risk factors for neurotoxicity following high-dose methotrexate may differ between ethnic groups.^8,12^ Biological differences may contribute to disparities in susceptibility to treatment-related toxicities. In addition, neighborhood-level social determinants of health are an emerging driver of disparities in ALL outcomes,^13,14^ which have not yet been evaluated in association with methotrexate-related toxicity.

There is an unmet need to reliably identify patients with ALL who are at greatest risk of methotrexate-related neurotoxicity and who may benefit most from neuroprotective strategies. Machine learning techniques present an opportunity to identify risk factors and provide accurate classification predictions in complex settings with extremely rare outcomes due to their non-parametric backbone and ability to handle unbalanced, high-dimensional, complex data.^15,16^ Artificial intelligence and machine learning (AI/ML) techniques, such as random forest (RF), are increasingly being used to aid in the prediction of cancer incidence and improve prognostic risk stratification for various malignancies, including pediatric and adolescent cancers.^17-21^ In particular, AI/ML-assisted technology has demonstrated promise for breast cancer,^22^ colon cancer,^23^ and melanoma screening,^24^ in some cases leading to Food and Drug Administration approval.^25^ While a recent study by Zhan et al.^16^ utilized machine learning algorithms to predict neutropenia and fever in children with B-cell ALL, AI/ML techniques have received relatively limited attention in adverse event prediction in pediatric oncology. Given the need to improve neurotoxicity risk assessment, we leveraged a demographically diverse cohort of children and adolescents diagnosed with ALL to evaluate whether clinical, sociodemographic, and area-based information may be used to reliably predict methotrexate-related neurotoxicity using machine learning and advanced statistical techniques.

## Methods

### Study population

The Reducing Ethnic Disparities in Acute Leukemias (REDIAL) Consortium was established in 2016 and currently operates at 6 pediatric cancer treatment centers in Texas (Texas Children’s Cancer Center (Houston), Vannie E. Cook Jr. Children’s Cancer Center (McAllen), Cook Children’s Medical Center (Forth Worth), Children’s Hospital of San Antonio (San Antonio), Children’s Medical Center at UT Southwestern [Dallas]) and California (Children’s Hospital of Orange County [Orange]). The Consortium includes both retrospective medical record review (patients diagnosed prior to 2016) and prospective enrollment and data collection (patients diagnosed in 2016 and after) of patients diagnosed with acute leukemia at each participating site. Institutional review boards at each site reviewed and approved the study protocol. When appropriate, informed consent and/or assent was obtained from patients and/or legal guardians.

This evaluation included REDIAL participants newly diagnosed between 2005 and 2019 with B- or T-cell ALL residing in Texas. Children diagnosed in California were excluded to ensure complete address history information. Eligible patients were diagnosed between 2 and 20 years of age and treated on or according to one of the following Children’s Oncology Group (COG) protocols: AALL0031, AALL0232, AALL0331, AALL0434, AALL07P4, AALL08P1, AALL1231, AALL1131, AALL0932, and AALL1122. Children with Down Syndrome, preexisting neurologic disorders, or insufficient treatment histories to determine methotrexate exposure (eg, missing protocol, arm, date of methotrexate therapy) were excluded.

### Data collection

Systematic chart reviews were performed by trained staff members at each REDIAL site to collect residential (ie, address at diagnosis) and clinical information, including: treatment protocol and arm, NCI risk group, ALL subtype, CNS status at diagnosis, cranial radiation therapy, maximum alanine (ALT) transaminase, aspartate (AST) transaminase, and bilirubin levels during induction, age at diagnosis, sex, self-reported race and ethnicity, and body mass index (BMI) at diagnosis. The severity of transaminitis and hyperbilirubinemia (ALT, AST, and bilirubin) were graded according to the Common Terminology Criteria for Adverse Event (CTCAE) guidelines (Version 5).^26^ Height and weight at diagnosis were recorded to calculate body mass index (kg/m^2^, BMI). Age- and gender-specific *z*-scores for BMI were generated using World Health Organization definitions for individuals <20 years of age or adult definitions if ≥20 years of age.^27^

#### Area-based social determinants of health

Addresses were geocoded and linked to census tracts, which describe small subdivisions of a county statistically designed to be homogenous in terms of key social measures, including economic status and living conditions. Census-tract level information information collected through the American Community Survey was accessed through the United States Census Bureau website to gather information on area-based social determinants of health. The American Community Survey releases census-tract-level data in 5-year increments. We assigned each child to area-based social determinants of health data based on residence at diagnosis with the American Community Survey timepoint closest to the date of diagnosis.

Using the linked American Community Survey data, the Yost socioeconomic status (SES) Index^28^ was calculated for each census tract. The Yost SES Index^28^ is a composite measure commonly used in cancer-based studies (eg, by SEER and the NCI) and is generated from a principal components analysis integrating information from 7 domains: median house value, median rent, median household income, the percentage living below 150% poverty line, education index, percentage working class, and percentage unemployed. Ultimately, only one component is retained based on the Kaiser criterion and a scree plot; the value is transformed from a scale of 1-5, with 1 indicating the highest SES and 5 indicating the lowest SES. For this study, the Yost Index was modeled continuously.

Latino enclaves are important social measures often used in epidemiologic studies of cancer. They are identified by a principal components analysis of the proportion of 4 different categories of people living within an area: Latino residents, foreign-born Latino residents, residents with limited English proficiency and who speak Spanish, and linguistically isolated households that speak Spanish.^29^ We calculated scores for each census tract. These scores were then grouped into statewide quintiles, from 1 (least ethnically distinct) to 5 (most ethnically distinct). We defined Latino enclaves as all tracts in the highest quintile.

#### Methotrexate exposure

IV and IT methotrexate doses were abstracted from electronic health records. IV methotrexate was classified as ≥1 g/m^2^ or <1 g/m^2^. If complete methotrexate dose data was unavailable, IV methotrexate dose was estimated from the post-induction treatment protocol and arm (Supplementary Table S1).

#### Neurotoxicity case identification

All brain magnetic resonance images conducted during ALL therapy were identified and manually reviewed for potential neurotoxicity. Relevant data were abstracted for each MRI, including the reason for the MRI, MRI findings, presentation of neurologic symptoms, date of symptom onset, prior IT and IV methotrexate exposure, and subsequent modifications to methotrexate therapy. Potential cases presented with symptoms consistent with methotrexate-related neurotoxicity (eg, seizure, stroke-like symptoms, altered mental status), within 21 days of the last IT or IV methotrexate infusion as well as evidence of neurotoxicity in the MRI including changes in the deep white matter which indicate leukoencephalopathy, oval-shaped lesions in the subcortical white matter, and hyperintensity in T2-weighted images. This definition has been used in previous publications and is consistent with a previous study which used the Delphi criteria.^7,8,10-12^ Each potential case was independently reviewed by 2 pediatric oncologists to determine whether the event was best classified as methotrexate-related neurotoxicity. All discrepancies between the first 2 independent reviewers were evaluated by a third pediatric oncologist to make a final determination.

#### Treatment phase

Dates for each phase of therapy were manually abstracted from electronic medical records (EMR)s. Events such as induction failure, refractory disease, and relapse often necessitate changes to ALL treatment protocols, which likely possess unique neurotoxicity risks. We limited our analysis to neurotoxic events occurring during the post-induction and pre-maintenance phases of therapy (consolidation, interim maintenance I, delayed intensification, and interim maintenance II) because: (1) the majority of neurotoxic events occur during these phases, and (2) the majority of refractory and relapse events occur at end of induction therapy or during maintenance therapy.

### Statistical analysis

Appropriate descriptive statistics for continuous (eg, mean, standard deviation [SD]) and categorical (eg, frequency and proportion of the total) variables were calculated to describe the study population. We evaluated the association of 14 variables with neurotoxicity: sex (binary factor, reference: male), race (factor), ethnicity (Latino or non-Latino, reference: non-Latino), age at diagnosis (continuous), BMI *z*-score (continuous), ALL subtype (binary factor, reference: B-ALL), CNS status at Diagnosis (binary factor, reference: CNS 1/2), cranial radiation therapy (factor, reference: no cranial radiation therapy), Yost score (continuous), Latino enclave (binary factor, reference: no enclave), peak ALT at induction (binary factor, reference: grade 1/grade 2), peak AST at induction (binary factor, reference: grade 1/grade 2), peak bilirubin at induction (binary factor, reference: grade 1/grade 2), methotrexate dose (binary factor, reference: <1 g/m^2^). Prior to RF and least absolute shrinkage and selection operator (LASSO) modeling, clinical, sociodemographic, and area-based information were imputed using the “missForest” package in R (R Core Team, Vienna, Austria) for any variables with <15% missingness as RF models cannot be built on datasets that contain any missingness. In addition, while participants and variables with high missingness rates (10% and 15%, respectively) were excluded from analysis, in the interest of maintaining power for these advanced modeling techniques, we opted for data imputation. No individuals were excluded due to >15% missing data. The missForest imputation package was selected based on its superior performance compared to other advanced imputation techniques.^21^ Variables with >15% missingness were excluded from all multivariable modeling; this was limited to ALT, AST, and bilirubin. All other variables were included in the multivariable models. Individuals with >15% missingness on any variables of interest were excluded.

Logistic regression was used to evaluate the unadjusted associations between each covariate of interest and neurotoxicity. A multivariable model was then constructed, taking all variables with *P* < .2 from the crude logistic regression. Odds ratios (ORs) and 95% confidence intervals (CIs) were reported for each crude association. Statistical significance was set at *P* < .05.

To construct a robust predictive model, we evaluated the performance of both RF^30^ and logistic-based LASSO^31^ techniques. Prior to model construction, data were randomly split with 80% of the observations used in the training stage and 20% withheld for the testing stage. We evaluated 4 different RF techniques: (1) RF without downsampling; (2) RF with downsampling; (3) RF with boosting; (4) RF with downsampling and boosting. Each forest was grown using 5-repeat, 10-fold cross-validation to optimize the tuning parameters. Models without boosting were set to 2000 trees with a tune length of 5. Ultimately, the RF model with downsampling and boosting showed superior performance when compared to the basic RF, RF with boosting, and the RF with downsampling (Supplementary Figure S1). Specifically, while the downsampled model performed similarly to the model with boosting and downsampling, the boosted model had higher sensitivity, correctly identifying a higher proportion of the neurotoxic cases and a lower test error rate, which suggests it may perform better in other populations. Therefore, the boosted RF with downsampling was selected. The LASSO model was built using downsampling with 5-repeat, 10-fold cross-validation to optimize the λ value to arrive at a consensus-trained model. The LASSO model incorporated all covariates of interest and all possible interaction terms.

The performance of the RF and LASSO models was compared to select the superior predictive model. We evaluated the sensitivity and specificity, test and train error rates, AUC, and ROC (in order of importance for model comparison) to select the superior model. Secondary to evaluating model predictive accuracy, risk factors were identified and described based on the superior model (ie, ORs and 95% CIs for LASSO or variable importance, which ranks how much the model contributed to the prediction based on the out-of-bag accuracy, for RF). Finally, given the reported disparities in neurotoxicity susceptibility,^8,12^ we performed a stratified analysis using our superior technique to evaluate whether the performance of our model and selected risk factors differed by ethnicity. The stratified models were limited to variables selected in the model (significant in LASSO or variable importance >0 for RFs). Due to sample size constrictions, stratified models were evaluated with an 85/15 random train/test split. All analyses were performed in R version 4.2.0 (R Core Team, Vienna, Austria) using the “randomForest,” “glmnet,” “tidyr,” “MLeval,” and “caret” packages.

## Results

Records were reviewed for 1348 total patients and a total of 128 cases were identified. Seven cases with a history of neurotoxicity during induction therapy were excluded and 5 cases with a history of neurotoxicity occurring during maintenance therapy were excluded. One case was excluded as the phase of the event was not available. Ten additional patients with incomplete documentation to determine whether a neurological event was linked to neurotoxicity were also excluded from the cohort. A total of 1325 patients were eligible and included in this analysis. Overall, the study population was 52.5% Latino, 45.1% female, with a mean age of 7 years at diagnosis (Table 1). The majority of the study population (76.6%) was diagnosed before 2016. About one-third of children resided in a Latino enclave at diagnosis and 19.8% resided in areas of the lowest SES. Nearly 9% (*n* = 115, 8.7%) of all patients experienced an acute neurotoxic event. The majority of acute neurotoxic events occurred during consolidation or interim maintenance I (*n* = 83) with 70% of the cases treated with protocols containing ≥1 g/m^2^ of methotrexate. Seventy-four percent of children who experienced neurotoxicity self-report as Latino. Most cases presented with stroke-like symptoms (*n* = 68), followed by seizure (*n* = 27), altered mental status (*n* = 10), tremors/numbness (*n* = 5), aphasia (*n* = 2), word-finding difficulty (*n* = 2), and syncopal episode (*n* = 1).

**Table 1.** Cohort clinical and demographic characteristics, by neurotoxicity case and control status.

	Neurotoxicity status		
	Control	Case	Total
	(*N* = 1210)	(*N* = 115)	(*N* = 1325)
Age at diagnosis (years)			
Mean (SD)	6.6 (±4.4)	11 (±4.8)	6.9 (±4.6)
Sex			
Female	544 (45.0%)	53 (46.1%)	597 (45.1%)
Male	666 (55.0%)	62 (53.9%)	728 (54.9%)
Self-reported race			
Asian	63 (5.2%)	1 (0.9%)	64 (4.8%)
Black	103 (8.5%)	8 (7.0%)	111 (8.4%)
Native American	32 (2.6%)	12 (10.4%)	44 (3.3%)
Other/unknown	92 (7.6%)	1 (0.9%)	93 (7.0%)
White	919 (76.0%)	93 (80.9%)	1012 (76.4%)
Missing	1 (0.1%)	0 (0%)	1 (0.1%)
Self-reported ethnicity			
Latino	610 (50.4%)	85 (73.9%)	695 (52.5%)
Non-Latino	600 (49.6%)	30 (26.1%)	630 (47.5%)
Yost index^1^			
1	218 (18.0%)	24 (20.9%)	242 (18.3%)
2	262 (21.7%)	19 (16.5%)	281 (21.2%)
3	212 (17.5%)	18 (15.7%)	230 (17.4%)
4	221 (18.3%)	25 (21.7%)	246 (18.6%)
5	237 (19.6%)	25 (21.7%)	262 (19.8%)
Missing	60 (5.0%)	4 (3.5%)	64 (4.8%)
Latino enclave			
Not residence in a Latino enclave	763 (63.1%)	65 (56.5%)	828 (62.5%)
Residence in a Latino enclave	398 (32.9%)	48 (41.7%)	446 (33.7%)
Missing	49 (4.0%)	2 (1.7%)	51 (3.8%)
BMI z-score			
Mean (SD)	0.58 (± 1.4)	1.1 (± 1.6)	0.63 (± 1.4)
Missing	141 (11.7%)	5 (4.3%)	146 (11.0%)
NCI risk group			
High risk	488 (40.3%)	86 (74.8%)	574 (43.3%)
Standard risk	689 (56.9%)	25 (21.7%)	714 (53.9%)
Missing	33 (2.7%)	4 (3.5%)	37 (2.8%)
ALL subtype			
B-ALL	1098 (90.7%)	99 (86.1%)	1197 (90.3%)
T-ALL	112 (9.3%)	16 (13.9%)	128 (9.7%)
CNS status at diagnosis			
CNS 1/2	1137 (94.0%)	106 (92.2%)	1243 (93.8%)
CNS 3	32 (2.6%)	4 (3.5%)	36 (2.7%)
Missing	41 (3.4%)	5 (4.3%)	46 (3.5%)
Methotrexate dosing regimen			
≥1 g/m^2^	510 (42.1%)	81 (70.4%)	591 (44.6%)
<1 g/m^2^	657 (54.3%)	27 (23.5%)	684 (51.6%)
Missing	43 (3.6%)	7 (6.1%)	50 (3.8%)
Cranial radiation therapy exposure			
No	1087 (89.8%)	106 (92.2%)	1193 (90.0%)
Yes	95 (7.9%)	9 (7.8%)	104 (7.8%)
Missing	28 (2.3%)	0 (0%)	28 (2.1%)
Diagnosis year			
Prior to 2016	936 (77.4%)	79 (68.7%)	1015 (76.6%)
2016 or later	274 (22.6%)	36 (31.3%)	310 (23.4%)
Maximum ALT during induction			
Grade 1/Grade 2	531 (43.9%)	60 (52.2%)	591 (44.6%)
Grade 3/Grade 4	98 (8.1%)	19 (16.5%)	117 (8.8%)
Missing	581 (48.0%)	36 (31.3%)	617 (46.6%)
Maximum AST during induction			
Grade 1/Grade 2	410 (33.9%)	57 (49.6%)	467 (35.2%)
Grade 3/Grade 4	38 (3.1%)	9 (7.8%)	47 (3.5%)
Missing	762 (63.0%)	49 (42.6%)	811 (61.2%)
Maximum total bilirubin during induction			
Grade 1/Grade 2	264 (21.8%)	27 (23.5%)	291 (22.0%)
Grade 3/Grade 4	19 (1.6%)	7 (6.1%)	26 (2.0%)
Missing	927 (76.6%)	81 (70.4%)	1008 (76.1%)

Abbreviations: ALT, alanine transaminase; ALL, acute lymphoblastic leukemia; AST, aspartate transaminase; BMI, body mass index; CNS, central nervous system; NCI, national cancer institute; SD, standard deviation.

^1^Low scores indicate most affluent and high scores indicate most deprived neighborhoods.

In unadjusted logistic models, we identified significant associations between neurotoxicity and older age at diagnosis (OR = 1.19, 95% CI: 1.15-1.24), ethnicity (OR = 2.79, 95% CI: 1.83-4.35, reference: non-Latino), exposure to ≥1 g/m^2^ of methotrexate (OR = 3.40, 95% CI: 2.25-5.25, reference: <1 g/m^2^), BMI *z*-score at diagnosis (OR = 1.33, 95% CI: 1.16-1.54), and maximum bilirubin during induction (OR = 3.60, 95% CI: 1.31-9.04; reference: grade 1/grade 2; Table 2). We also identified a suggestive, but not statistically significant, association between neurotoxicity and residence in a Latino enclave (OR = 1.39, 95% CI = 0.94-2.04, *P*-value = .10). Similarly, we identified a suggestive association with ALL subtype, where T-cell ALL is associated with a slightly higher risk for neurotoxity (OR = 1.58, 95% CI = 0.87-2.71, *P*-value = .11). It is worth noting here the small proportion of T-ALL (9.7%) versus B-ALL (90.3)% patients in our cohort. In the multivariable model, age at diagnosis (OR = 1.16, 95%CI: 1.11-1.21), ethnicity (OR = 2.80, 95% CI: 1.73-4.61), and exposure to ≥1 g/m^2^ of methotrexate (OR = 1.73, 95% CI: 1.07-2.83) remained statistically significant.

**Table 2.** Association between neurotoxicity and each of the covariates of interest estimated using crude and multivariable logistic regression.

Variable	Odds ratio (95% CI)	*P*-value
* Crude models*		
Age at diagnosis	1.19 (1.15, 1.24)	<0.001
Sex		
Male		
Female	1.05 (0.71, 1.54)	0.816
Ethnicity		
Non-Latino		
Latino	2.79 (1.83, 4.35)	<0.001
Race		
White		
Black	0.77 (0.34, 1.53)	0.491
Asian	0.16 (0.01, 0.72)	0.068
Native	3.71 (1.78, 7.27)	<0.001
Other/Unknown	0.11 (0.01, 0.49)	0.027
Methotrexate dosing regimen		
<1g/m^2^		
≥1 g/m^2^	3.40 (2.25, 5.25)	<0.001
ALL Subtype		
B-ALL		
T-ALL	1.58 (0.87, 2.71)	0.109
CNS status at diagnosis		
CNS 1/CNS 2 (−)		
CNS 3 (+)	1.33 (0.39, 3.42)	0.6
BMI *z*-score at diagnosis	1.33 (1.16, 1.54)	<0.001
Exposure to cranial radiation therapy		
No		
Yes	1.00 (0.46, 1.93)	0.992
Yost Index	1.05 (0.91, 1.20)	0.519
Residence in a Latino Enclave		
No		
Yes	1.39 (0.94, 2.04)	0.099
Max AST during induction		
Grade 1/Grade 2		
Grade 3/Grade 4	1.70 (0.74, 3.57)	0.179
Max ALT during induction		
Grade 1/Grade 2		
Grade 3/Grade 4	1.72 (0.96, 2.95)	0.058
Max bilirubin during induction		
Grade 1/Grade 2		
Grade 3/Grade 4	3.60 (1.31, 9.04)	0.008
* Multivariable* ^1^ *model*		
Age at diagnosis	1.16 (1.11, 1.21)	<0.01
Ethnicity		
Non-Latino		
Latino	2.80 (1.73, 4.61)	<0.01
Methotrexate dosing regimen		
<1 g/m^2^		
≥1 g/m^2^	1.73 (1.07, 2.83)	0.028
ALL subtype		
B-ALL		
T-ALL	1.43 (0.76, 2.55)	0.247
BMI *z*-score at diagnosis	1.13 (0.97, 1.31)	0.116
Residence in a Latino Enclave		
No		
Yes	0.86 (0.55, 1.33)	0.487

Abbreviations: ALT, alanine transaminase; ALL, acute lymphoblastic leukemia; AST, aspartate transaminase; BMI, body mass index; CI, confidence interval; CNS, central nervous system.

^1^Multivariable model includes all variables with *P* < .20 in crude models.

**Figure 1.**
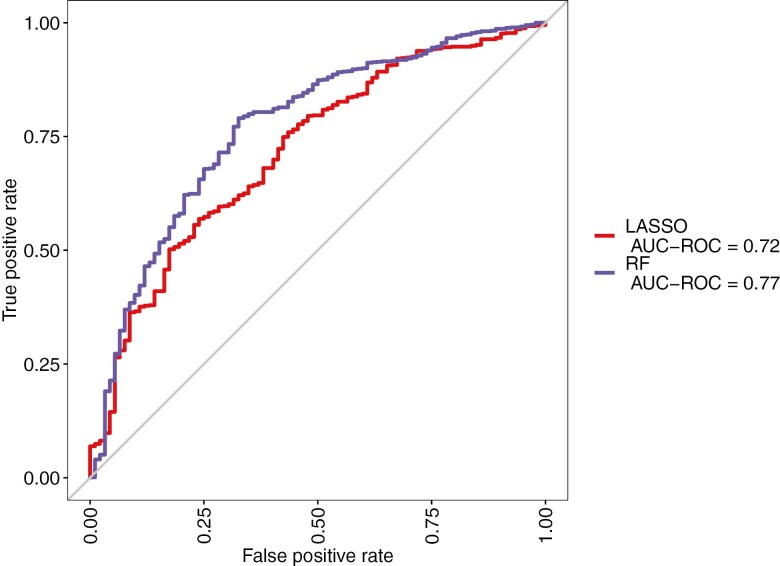
Comparative ROC plot of random forest (RF) versus LASSO modeling techniques.

The RF model outperformed the LASSO in all model performance metrics (Table 3 and Figure 1). Specifically, the RF had a substantially lower test error rate (0.24 vs. 0.40); the lower test error rate is especially important given the goal to extend risk prediction to other populations of children diagnosed with ALL. Additionally, the RF had a higher sensitivity, correctly identifying 73% of the cases whereas the LASSO correctly identified 67% of the cases. The RF also had a higher AUC (0.77 vs. 0.72), lower training error rate (0.29 vs. 0.31), and higher sensitivity/specificity values (0.72/0.69 vs. 0.67/0.60).

**Table 3.** Comparative random forest (with downsampling and boosting) and LASSO model performance statistics.

	Train error	Test error	AUC	Sensitivity	Specificity
**Random forest**	0.291	0.242	0.770	0.730	0.685
**LASSO**	0.312	0.398	0.720	0.674	0.598

**Figure 2.**
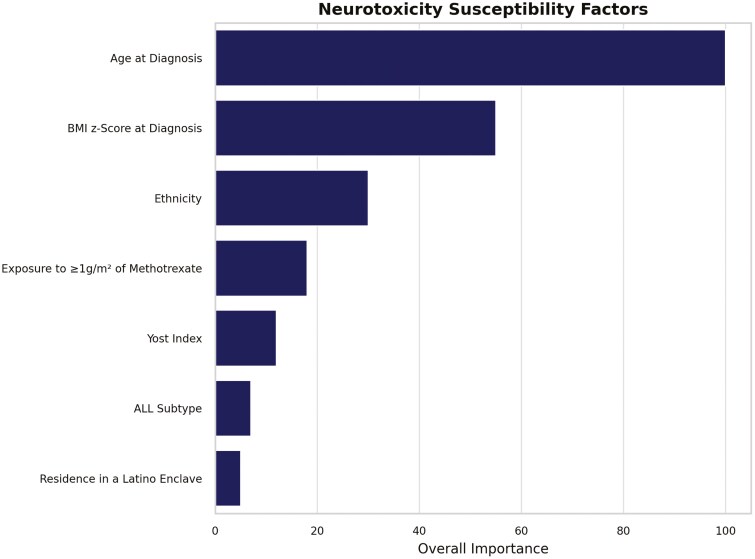
Variable importance plot for identified neurotoxicity susceptibility factors (variable importance >0).

The RF model indicated several factors influencing neurotoxicity susceptibility, measured by variable importance (scaled: 0-100). Age at diagnosis was the most important predictor (importance = 100%), followed by BMI *z*-score at diagnosis (importance = 32.9%), ethnicity (importance = 17.0%), exposure to ≥1 g/m^2^ of methotrexate (importance = 9.7%), Yost index (importance = 5.1%), ALL subtype (importance = 4.8%), and residence in a Latino enclave (importance = 3.9%; Figure 2). When stratified by ethnicity, the model demonstrated similar performance in both ethnic groups with minimal differences observed in variable importance assigned to each factor (Supplementary Table S2 and Figure S2).

## Discussion

Here, we developed a novel risk prediction model for methotrexate-related neurotoxicity using data from a large cohort of children with ALL from diverse population groups. Our approach leveraged machine learning approaches to classify post-induction neurotoxicity risk based on a set of observed clinical and sociodemographic factors. Consistent with previous studies, we found methotrexate-related neurotoxicity occurred most frequently in children diagnosed with ALL at older ages and in Latino children.^7-9,12^ We further evaluated potential disparities in neurotoxicity across area-based social determinants of health and found some evidence that residential factors may impact toxicity susceptibility among children undergoing therapy for ALL.

Previous studies have indicated exposure to high-dose methotrexate, ethnicity, and age at diagnosis as being associated with neurotoxicity, with age at diagnosis being the most well-demonstrated association.^7-9,12^ Similarly, our study identified older age at diagnosis as one of the most significant (logistic models) and important (RF) factors. Methotrexate dosing ≥1 g/m^2^ was also a highly significant (*P* < .01) and important predictor in our study and was associated with a nearly two-fold (OR = 1.73, 95% CI: 1.07-2.82) odds of neurotoxicity compared to doses <1 g/m^2^ in adjusted logistic models. Additionally, our study suggested some similar recently reported associations with bilibrubin levels during induction therapy^9^ in crude models. However, due to limited data availability (>15% missingness rate), we were unable to evaluate the lab measures (AST, ALT, and bilirubin) in multivariable models. Additionally, we did not observe significant associations with AST or ALT to evaluate associations with transaminitis. Finally, consistent with our previous studies suggesting ethnic disparities in neurotoxicity risk,^8,12^ we also found that Latino children were at a nearly 3-fold increased risk for neurotoxicity (OR = 2.79, 95% CI: 1.83-4.35), when compared to their non-Latino counterparts, a disparity which remained after accounting for other clinical factors in multivariable models.

Notably, this study was the first to evaluate area-based social determinants of health related to neurotoxicity. In crude analyses, we found a suggestive association between residence in a Latino enclave and neurotoxicity (OR = 1.39, *P*-value = .10) with the association remaining in RF models (among the top 10 most important predictors). However, area-based social determinants of health were not significantly associated with neurotoxicity in multivariable logistic regression after accounting for other clinical and demographic variables. Previously, residence in a Latino enclave and other composite measures of area-based socioeconomics, such as area deprivation index, were demonstrated to be associated with lower overall survival among children diagnosed with ALL.^13,14^ Considering the suggestive associations we observed between these factors and neurotoxicity and their previously reported association with survival, area-based social determinants of health deserve further consideration in future investigations of treatment outcomes among children undergoing ALL therapy.

Strengths of our study include the use of advanced statistical techniques to develop a preliminary neurotoxicity prediction model in a multi-site, ethnically and geographically diverse population. Notable strengths of this study are the number of neurotoxicity cases included and the centralized approach to reviewing clinical records and neurotoxicity classification across all sites. In addition, we leveraged, evaluated, and compared the performance of 2 advanced modeling techniques in this study. Still, our study should be considered in light of a few limitations. First, data collected were retrospective and thus limited by information available in the EMR. Therefore we were unable to include genomic or molecular information, for example, in our models. Second, there may be some time-to-event and censoring of observations, complexities that were not accounted for using the LASSO and RF techniques. However, events such as death or relapse often occur in maintenance therapy or post-therapy, while we restricted our analysis to the post-induction/pre-maintenance phases of therapy when neurotoxic events most frequently occur. In our cohort, 90% (*n* = 115) of all events occurred during post-induction, with 123 out of the 128 total events occurring before maintenance. As a result, our model may not perform well at predicting neurotoxic events occurring in the induction or maintenance phases of therapy. Third, we were not able to perform any external validation of our predictive model in other cohorts.

This is the first advanced analytic approach taken to understanding neurotoxicity risk among children diagnosed with ALL. We demonstrate that methotrexate-related neurotoxicity is a reasonably predictable outcome during ALL post-induction/pre-maintenance when considering key clinical, demographic, and area-based information. While the model we developed gave a reasonable predictive performance (test error rate 24%), this study highlighted the potential for machine learning to identify children at risk for neurotoxicity as well as pointed to novel potential risk factors for an insufficiently studied outcome. Additional research is needed to externally validate the performance of our model in other populations, replicate identified associations, and assess whether other sources of variability, including pharmacogenomic information, can improve model performance. The clinical management of methotrexate-related neurotoxicity poses a challenge in children with ALL,^32^ with current approaches involving symptom management and rechallenge with methotrexate after neurologic recovery,^32,33^ often with folinic acid rescue.^11,34^ Therefore, a validated neurotoxicity risk prediction model may prospectively inform the delivery of methotrexate therapy and/or supportive care to at-risk children prior to the development of toxicity (eg, individualized methotrexate dosing or administering earlier folinic acid rescue).^35,36^ We anticipate that the results from this study may help guide the development of personalized treatment strategies to reduce the burden of neurotoxicity and improve health outcomes in children undergoing therapy for ALL.

## Supplementary Material

oyaf055_suppl_Supplementary_Tables_S1-S2_Figures_S1-S2

## Data Availability

The datasets generated during and/or analyzed during the current study are available from the corresponding author on reasonable request.

## References

[1] American Cancer Society [Internet]. 2019. Survival Rates for Childhood Leukemias. Available from: https://www.cancer.org/cancer/types/cancer-in-children/key-statistics.html

[2] Lupo PJ , Spector LG. Cancer progress and priorities: childhood cancer. Cancer Epidemiol Biomarkers Prev. 2020;29:1081-1094. https://doi.org/10.1158/1055-9965.EPI-19-0941 32482635 PMC9400945

[3] Linabery AM , Ross JA. Trends in childhood cancer incidence in the U.S. (1992-2004). Cancer. 2008;112:416-432. https://doi.org/10.1002/cncr.23169 18074355

[4] Hunger SP , Lu X, Devidas M, et al Improved survival for children and adolescents with acute lymphoblastic leukemia between 1990 and 2005: a report from the children’s oncology group. J Clin Oncol. 2012;30:1663-1669. https://doi.org/10.1200/JCO.2011.37.8018 22412151 PMC3383113

[5] Gupta S , Dai Y, Chen Z, et al Racial and ethnic disparities in childhood and young adult acute lymphocytic leukaemia: secondary analyses of eight Children’s Oncology Group cohort trials. Lancet Haematol. 2023;10:e129-e141. https://doi.org/10.1016/S2352-3026(22)00371-4 36725118 PMC9951049

[6] Larsen EC , Devidas M, Chen S, et al Dexamethasone and high-dose methotrexate improve outcome for children and young adults with high-risk B-acute lymphoblastic leukemia: a report from children’s oncology group study AALL0232. J Clin Oncol: Off J Am Soc Clin Oncol. 2016;34:2380-2388. https://doi.org/10.1200/JCO.2015.62.4544 PMC4981974 27114587

[7] Bhojwani D , Sabin ND, Pei D, et al Methotrexate-induced neurotoxicity and leukoencephalopathy in childhood acute lymphoblastic leukemia. J Clin Oncol: Off J Am Soc Clin Oncol. 2014;32:949-959. https://doi.org/10.1200/JCO.2013.53.0808 PMC3948096 24550419

[8] Taylor OA , Brown AL, Brackett J, et al Disparities in neurotoxicity risk and outcomes among pediatric acute lymphoblastic leukemia patients. Clin Cancer Res: Off J Am Assoc Cancer Res. 2018;24:5012-5017. https://doi.org/10.1158/1078-0432.CCR-18-0939 PMC6191323 30206159

[9] Mateos MK , Marshall GM, Barbaro PM, et al Methotrexate-related central neurotoxicity: clinical characteristics, risk factors and genome-wide association study in children treated for acute lymphoblastic leukemia. Haematologica. 2021;107:635-643. https://doi.org/10.3324/haematol.2020.268565 PMC8883571 33567813

[10] Schmiegelow K , Attarbaschi A, Barzilai S, et al; Ponte di Legno toxicity working group. Consensus definitions of 14 severe acute toxic effects for childhood lymphoblastic leukaemia treatment: a Delphi consensus. Lancet Oncol. 2016;17:e231-e239. https://doi.org/10.1016/S1470-2045(16)30035-3 27299279

[11] Mahoney DHJ , Shuster JJ, Nitschke R, et al Acute neurotoxicity in children with B-precursor acute lymphoid leukemia: an association with intermediate-dose intravenous methotrexate and intrathecal triple therapy--a Pediatric Oncology Group study. J Clin Oncol. 1998;16:1712-1722. https://doi.org/10.1200/JCO.1998.16.5.1712 9586883

[12] Harris RD , Bernhardt MB, Zobeck MC, et al Ethnic-specific predictors of neurotoxicity among patients with pediatric acute lymphoblastic leukemia after high-dose methotrexate. Cancer. 2023;129:1287-1294. https://doi.org/10.1002/cncr.34646 36692972 PMC10625847

[13] Schraw JM , Peckham-Gregory EC, Hughes AE, et al Residence in a hispanic enclave is associated with inferior overall survival among children with acute lymphoblastic leukemia. Int J Environ Res Public Health. 2021;18:9273. https://doi.org/10.3390/ijerph18179273 34501862 PMC8430860

[14] Schraw JM , Peckham-Gregory EC, Rabin KR, et al Area deprivation is associated with poorer overall survival in children with acute lymphoblastic leukemia. Pediatr Blood Cancer. 2020;67:e28525. https://doi.org/10.1002/pbc.28525 32573920

[15] Cafri G , Li L, Paxton EW, Fan J. Predicting risk for adverse health events using random forest. J Appl Stat. 2018;45:2279-2294. https://doi.org/10.1080/02664763.2017.1414166

[16] Zhan M , Chen ZB, Ding CC, et al Machine learning to predict high-dose methotrexate-related neutropenia and fever in children with B-cell acute lymphoblastic leukemia. Leuk Lymphoma. 2021;62:2502-2513. https://doi.org/10.1080/10428194.2021.1913140 33899650

[17] Huang B , Tian S, Zhan N, et al Accurate diagnosis and prognosis prediction of gastric cancer using deep learning on digital pathological images: a retrospective multicentre study. EBioMedicine. 2021;73:103631. https://doi.org/10.1016/j.ebiom.2021.103631. 34678610 PMC8529077

[18] Chang CC , Chen CH, Hsieh JG, Jeng JH. Iterated cross validation method for prediction of survival in diffuse large B-cell lymphoma for small size dataset. Sci Rep. 2023;13:1438. https://pubmed.ncbi.nlm.nih.gov/36697456/36697456 10.1038/s41598-023-28394-6 PMC9876907

[19] Lian J , Deng J, Hui ES, et al Early stage NSCLS patients’ prognostic prediction with multi-information using transformer and graph neural network model. Elife. 2022;11:e80547. https://pubmed.ncbi.nlm.nih.gov/36194194/36194194 10.7554/eLife.80547 PMC9531948

[20] Bhambhvani HP , Zamora A, Velaer K, Greenberg DR, Sheth KR. Deep learning enabled prediction of 5-year survival in pediatric genitourinary rhabdomyosarcoma. Surg Oncol. 2021;36:23-27. https://doi.org/10.1016/j.suronc.2020.11.002. 33276260

[21] Li W , Dong Y, Liu W, et al A deep belief network-based clinical decision system for patients with osteosarcoma. Front Immunol. 2022;13:1003347. https://pubmed.ncbi.nlm.nih.gov/36466868/36466868 10.3389/fimmu.2022.1003347 PMC9716099

[22] Schaffter T , Buist DSM, Lee CI, et al; and the DM DREAM Consortium. Evaluation of combined artificial intelligence and radiologist assessment to interpret screening mammograms. JAMA Netw Open. 2020;3:e200265. https://doi.org/10.1001/jamanetworkopen.2020.0265. 32119094 PMC7052735

[23] Seager A , Sharp L, Neilson LJ, et al; COLO-DETECT trial team. Polyp detection with colonoscopy assisted by the GI Genius artificial intelligence endoscopy module compared with standard colonoscopy in routine colonoscopy practice (COLO-DETECT): a multicentre, open-label, parallel-arm, pragmatic randomised controlled trial. Lancet Gastroenterol Hepatol. 2024;9:911-923. https://doi.org/10.1016/S2468-1253(24)00161-4. 39153491

[24] Manolakos D , Patrick G, Geisse JK, et al Use of an elastic-scattering spectroscopy and artificial intelligence device in the assessment of lesions suggestive of skin cancer: a comparative effectiveness study. JAAD Int. 2023;14:52-58. https://doi.org/10.1016/j.jdin.2023.08.019. 38143790 PMC10746496

[25] Benjamens S , Dhunnoo P, Meskó B. The state of artificial intelligence-based FDA-approved medical devices and algorithms: an online database. NPJ Digit Med. 2020;3:118. https://doi.org/10.1038/s41746-020-00324-0. 32984550 PMC7486909

[26] Common Terminology Criteria for Adverse Events (CTCAE) Version 5.0 [Internet]. 2017. Available from: https://ctep.cancer.gov/protocoldevelopment/electronic_applications/docs/ctcae_v5_quick_reference_5x7.pdf

[27] 2000 CDC Growth Charts for the United States: Methods and Development 2010 [Internet]. 2010. Available from: http://www.cdc.gov/nchs/data/series/sr_11/sr11_246.pdf 12043359

[28] Yost K , Perkins C, Cohen R, Morris C, Wright W. Socioeconomic status and breast cancer incidence in California for different race/ethnic groups. Cancer Causes Control. 2001;12:703-711. https://doi.org/10.1023/a:1011240019516 11562110

[29] Gomez SL , Shariff-Marco S, DeRouen M, et al The impact of neighborhood social and built environment factors across the cancer continuum: Current research, methodological considerations, and future directions. Cancer. 2015;121:2314-2330. https://doi.org/10.1002/cncr.29345 25847484 PMC4490083

[30] Breiman L. Random Forests. Mach Learn. 2001;45:5-32. https://doi.org/10.1023/A:1010933404324

[31] Tibshirani R. The lasso method for variable selection in the cox model. Stat Med. 1997;16:385-395. https://doi.org/10.1002/(sici)1097-0258(19970228)16:4<385::aid-sim380>3.0.co;2-3 9044528

[32] Bhojwani D , Bansal R, Wayne AS. Managing therapy-associated neurotoxicity in children with ALL. Hematology Am Soc Hematol Educ Program. 2021;2021:376-383. https://doi.org/10.1182/hematology.2021000269 34889354 PMC8791096

[33] Badke C , Fleming A, Iqbal A, et al Rechallenging With Intrathecal Methotrexate After Developing Subacute Neurotoxicity in Children With Hematologic Malignancies. Pediatr Blood Cancer. 2016;63:723-726. https://doi.org/10.1002/pbc.25850 26681571

[34] Winick NJ , Bowman WP, Kamen BA, et al Unexpected acute neurologic toxicity in the treatment of children with acute lymphoblastic leukemia. J Natl Cancer Inst. 1992;84:252-256. https://doi.org/10.1093/jnci/84.4.252 1734087

[35] Foster JH , Bernhardt MB, Thompson PA, Smith EO, Schafer ES. Using a bedside algorithm to individually dose high-dose methotrexate for patients at risk for toxicity. J Pediatr Hematol Oncol. 2017;39:72-76. https://doi.org/10.1097/MPH.0000000000000696 27820134

[36] Foster JH , Thompson PA, Bernhardt MB, et al A prospective study of a simple algorithm to individually dose high-dose methotrexate for children with leukemia at risk for methotrexate toxicities. Cancer Chemother Pharmacol. 2019;83:349-360. https://doi.org/10.1007/s00280-018-3733-2 30488179

