# Supplementary material for: Prediction of methotrexate neurotoxicity using clinical, sociodemographic, and area-based information in children with acute lymphoblastic leukemia
Source: Oncologist. 2025 Jun 23;30(6):oyaf055. doi: 10.1093/oncolo/oyaf055 (PMC12205976; doi:10.1093/oncolo/oyaf055)

## Supplementary Material

**Table S1**. Table of rules for categorizing patients as $\geq$1 g/m^2^ or <1g/m^2^ for each post-induction treatment protocol and arm included in this study.

| **Protocol and Arm** | **Classification Rule** |
| --- | --- |
| AALL0031 |  |
| All Arms | ≥1 g/m^2^ |
| AALL0232 |  |
| DC | <1 g/m^2^  if before 2011 |
|  | ≥1 g/m^2^ if day 1 of maintenance 01-01-2011 or after |
| PC | <1 g/m^2^ if before 2011 |
|  | ≥1 g/m^2^ if day 1 of maintenance 01-01-2011 or after |
| DH | ≥1 g/m^2^ |
| PH | ≥1 g/m^2^ |
| AALL0331 |  |
| SR-Avg /IS-IV/SS-IV | <1 g/m^2^ |
| SR-Low/LRS-IV/LRAsp | <1 g/m^2^ |
| SR-High | <1 g/m^2^  if before 2011 |
|  | ≥1 g/m^2^ if day 1 of maintenance 01-01-2011 or after unless received CRT^1^ |
| AALL0434 |  |
| Arm A | <1 g/m^2^ |
| Arm B | ≥1 g/m^2^ |
| Arm C | ≥1 g/m^2^ |
| Arm D | ≥1 g/m^2^ |
| AALL07P4 |  |
| All Arms | ≥1 g/m^2^ if day 1 of IM1/2 or DI after 01-01-2011, unless received CRT |
|  | <1 g/m^2^ otherwise |
| AALL08P1 |  |
| All Arms | ≥1 g/m^2^ if day 1 of maintenance after 01-01-2011 |
|  | <1 g/m^2^ otherwise |
| AALL0932 |  |
| All Arms | <1 g/m^2^ |
| AALL1231 |  |
| Arm A Intermediate Risk/IR | ≥1 g/m^2^ |
| Arm A Standard Risk/SR | <1 g/m^2^ |
| Arm B Intermediate Risk/IR | ≥1 g/m^2^ |
| Arm B Standard Risk/SR | <1 g/m^2^ |
| AALL1131 |  |
| All Arms | ≥1 g/m^2^ |
| AALL1122 |  |
| All Arms | ≥1 g/m^2^ |

^1^Cranial radiation therapy

**Table S2**. Variable importance stratified by ethnicity.

| **Non-Latino** | | **Latino** | |
| --- | --- | --- | --- |
| **Variable** | **Overall Importance** | **Variable** | **Overall Importance** |
| Age at Diagnosis | 100.00 | Age at Diagnosis | 100.00 |
| BMI^1^ z-Score at Diagnosis | 91.50 | BMI^1^ z-Score at Diagnosis | 97.61 |
| Yost Index | 44.48 | Yost Index | 25.24 |
| Exposed to ≥1 g/m^2^ Methotrexate | 4.49 | Resides in a Latino Enclave | 5.66 |
| T-ALL^2^ | 0.00 | Exposed to ≥1 g/m^2^ Methotrexate | 4.57 |
| Resides in a Latino Enclave | 0.00 | T-ALL^2^ | 0.00 |

^1^ BMI = Body mass index

^2^ ALL = acute lymphoblastic leukemia

**Figure S1**. Comparative ROC plot of different random forest (RF), random forest with boosting (RF-B) random forest with boosting and downsampling (RF-B&D), random forest with downsampling (RF-D) techniques.


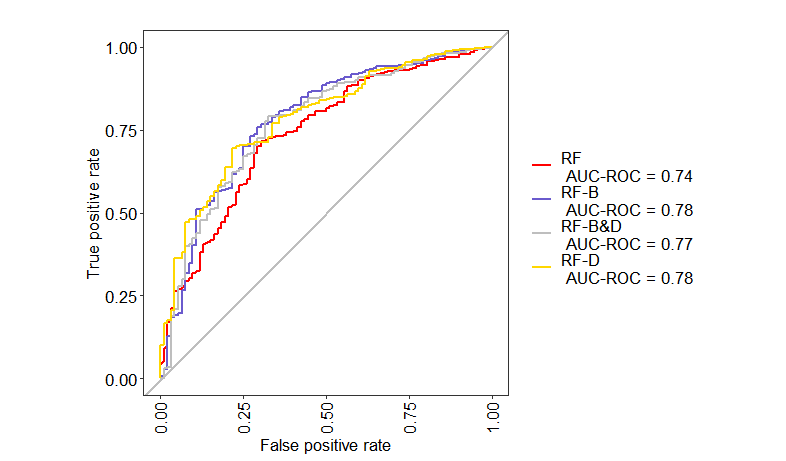


Figure Legend:

RF: Random Forest

RF-B: Boosted Random Forest

RF-B&D: Boosted Random Forest with Downsampling

RF-D: Random Forest with Downsampling

**Figure S2.** ROC plot of the random forest stratified by ethnicity.


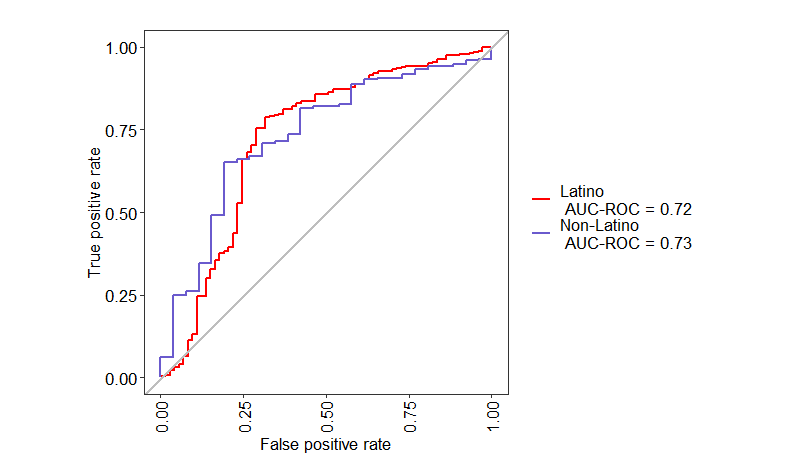

Supplement: oyaf055_suppl_Supplementary_Tables_S1-S2_Figures_S1-S2 [file oyaf055_suppl_supplementary_tables_s1-s2_figures_s1-s2.docx]
